# Protective Immunity against Gamma and Zeta Variants after Inactivated SARS-CoV-2 Virus Immunization

**DOI:** 10.3390/v13122440

**Published:** 2021-12-04

**Authors:** Marcilio Jorge Fumagalli, Luiza Antunes Castro-Jorge, Thais Fernanda de Campos Fraga-Silva, Patrick Orestes de Azevedo, Carlos Fabiano Capato, Bruna Amanda Cruz Rattis, Natália Satchiko Hojo-Souza, Vitor Gonçalves Floriano, Julia Teixeira de Castro, Simone Gusmão Ramos, Benedito Antônio Lopes da Fonseca, Vânia Luiza Deperon Bonato, Ricardo Tostes Gazzinelli, Luiz Tadeu Moraes Figueiredo

**Affiliations:** 1 Virology Research Center, Ribeirão Preto Medical School, University of São Paulo, Ribeirão Preto 14049-900, São Paulo, Brazil; luizacastro@gmail.com (L.A.C.-J.); fabianocapato@usp.br (C.F.C.); vitor.floriano@usp.br (V.G.F.); baldfons@fmrp.usp.br (B.A.L.d.F.); ltmfigue@fmrp.usp.br (L.T.M.F.); 2 Basic and Applied Immunology Program, Ribeirão Preto Medical School, University of São Paulo, Ribeirão Preto 14049-900, São Paulo, Brazil; vlbonato@fmrp.usp.br (V.L.D.B.); Ricardo.Gazzinelli@umassmed.edu (R.T.G.); 3 Department of Biochemistry and Immunology, Ribeirão Preto Medical School, University of São Paulo, Ribeirão Preto 14049-900, São Paulo, Brazil; thaisfragasilva@gmail.com; 4 Immunopathology Laboratory, René Rachou Institute, Oswaldo Cruz Foundation, Belo Horizonte 30190-002, Minas Gerais, Brazil; patrickoazevedo@gmail.com (P.O.d.A.); nataliasatchiko@gmail.com (N.S.H.-S.); juliatcastro@hotmail.com (J.T.d.C.); 5 Department of Pathology, Ribeirão Preto Medical School, University of São Paulo, Ribeirão Preto 14049-900, São Paulo, Brazil; bruna.rattis@usp.br (B.A.C.R.); sgramos@fmrp.usp.br (S.G.R.); 6 Platform of Translational Medicine, Fundação Oswaldo Cruz, Ribeirão Preto Medical School, University of São Paulo, Ribeirão Preto 14049-900, São Paulo, Brazil

**Keywords:** SARS-CoV-2, Gamma variant, Zeta variant, cross-protection, inactivated vaccine

## Abstract

The persistent circulation of SARS-CoV-2 represents an ongoing global threat due to the emergence of new viral variants that can sometimes evade the immune system of previously exposed or vaccinated individuals. We conducted a follow-up study of adult individuals that had received an inactivated SARS-CoV-2 vaccine, evaluating antibody production and neutralizing activity over a period of 6 months. In addition, we performed mice immunization with inactivated SARS-CoV-2, and evaluated the immune response and pathological outcomes against Gamma and Zeta variant infection. Vaccinated individuals produced high levels of antibodies with robust neutralizing activity, which was significantly reduced against Gamma and Zeta variants. Production of IgG anti-S antibodies and neutralizing activity robustly reduced after 6 months of vaccination. Immunized mice demonstrated cellular response against Gamma and Zeta variants, and after viral infection, reduced viral loads, IL-6 expression, and histopathological outcome in the lungs. TNF levels were unchanged in immunized or not immunized mice after infection with the Gamma variant. Furthermore, serum neutralization activity rapidly increases after infection with the Gamma and Zeta variants. Our data suggest that immunization with inactivated WT SARS-CoV-2 induces a promptly responsive cross-reactive immunity response against the Gamma and Zeta variants, reducing COVID-19 pathological outcomes.

## 1. Introduction

Severe acute respiratory syndrome coronavirus-2 (SARS-CoV-2) emerged in late 2019 in Wuhan, China [1]. SARS-CoV-2 is the causative agent of the coronavirus disease 2019 (COVID-19) pandemic, a human respiratory disease characterized by dry cough, headache, fever, severe pneumonia, and shortness of breath that can rapidly progress to respiratory failure and death [2]. SARS-CoV-2 has globally infected more than 200 million people, resulting in more than 4 million deaths, representing one of the most human life-threatening species of coronavirus [3]. SARS-CoV-2 belongs to the *Betacoronavirus* genus from the *Coronaviridae* family. It harbors a positive-sense single-stranded genomic RNA that encodes four structural proteins (spike (S), envelope (E), membrane (M), and nucleocapsid (N)), 16 nonstructural proteins (nsp1 to nsp16), and several accessory proteins [4]. Among these proteins, the S protein is the primary major antigen employed on vaccine development in many distinct platforms [5,6,7].

Multiple vaccines are being used to prevent COVID-19, including DNA- and RNA-based formulations, subunits containing viral epitopes, adenovirus-based vectors, and inactivated whole virus [8]. Among these platforms, inactivated-virus vaccines are generally safe and are traditionally used to prevent viral infections, such as for the influenza virus and poliovirus [9,10]. Many studies described the immunization effectiveness of the inactivated SARS-CoV-2 vaccine in human clinical trials [11,12,13,14,15]. Large-scale vaccination is globally occurring with the CoronaVac vaccine from Sinovac Life Sciences (China) [16], which utilizes inactivated whole SARS-CoV-2, with extensive distribution in more than 27 countries in Central and South America, Africa, Asia, and Europe [17]. Its safety, tolerability, and immunogenicity were evaluated in different human cohorts [13,18,19,20,21]. However, SARS-CoV-2 evolution is characterized by numerous mutations that can often lead to the emergence of new variants, which can change viral characteristics and compromise the immune response and vaccine effectiveness of a population, representing increased concern to health systems [22].

The Gamma (P.1) variant of SARS-CoV-2 may have emerged in Manaus (Brazil) in November 2020, has since been predominant in the country, and is considered to be a variant of concern (VOC) [23]. The Zeta variant (P.2) was identified in samples collected between April and November 2020 in the Rio de Janeiro state (Brazil) and was classified as a variant of interest (VOI) [24]. Although both the P.1 (20J/501Y.V3 variant) and P.2 (20J variant) lineages are descended from B.1.28 and share the E484K mutation, the two variants emerged independently in different epidemiological contexts [24]. One of the hypotheses of the abrupt increase in numbers of COVID-19 hospital admissions in early 2021 in Brazil is attributed to immune evasion by these new variants [25]. A study conducted in Brazil showed that the P.1 lineage acquired 17 mutations on the S protein, including K417T, E484K, and N501Y, which were associated with higher infectivity and transmissibility [23]. Comparatively, the P.2 lineage only exhibits the E484K mutation [24]. Many studies described that mutations in the SARS-CoV-2 variants, including Gamma and Zeta, are responsible for conferring resistance against neutralizing antibodies in individuals who had been vaccinated by ChAdOx1 (adenoviral vector), mRNA-1273, and BNT162b2 (mRNA) or CoronaVac vaccines [26,27,28,29,30]. Studies investigating cellular immune response demonstrated that T-cell activation in individuals vaccinated by mRNA vaccines (Moderna or Pfizer/BioNTech) is not significantly affected by mutations found in the Alpha (B.1.1.7 UK), Beta (B.1.351 South Africa), Gamma, and Epsilon (B.1.427 CA, USA) variants [31,32]. In contrast, another study with BNT162b2-vaccinated individuals demonstrated that T-cell response against SARS-CoV-2 variants might be increased, abrogated, or unchanged depending on the host HLA polymorphisms [33]. In any case, the clinical impact of viral resistance to neutralization and changes in the cellular immune response against SARS-CoV-2 variants in vaccinated individuals remain not fully understood. 

SARS-CoV-2 pathogenesis was evaluated using several different animal models, such as mice, Syrian hamsters, ferrets, and nonhuman primates [34]. All these species except mice are susceptible to infection with SARS-CoV-2. Hamsters, ferrets, and nonhuman primates develop mild illness and recover spontaneously [35]. The mouse model could be suitable for a preliminary evaluation of vaccine candidates and therapeutic agents for COVID-19, but common laboratory mouse strains are not promptly infected by WT SARS-CoV-2 due to poor compatibility binding interactions between the murine ACE2 receptor and viral spike protein [36]. However, the SARS-CoV-2 Beta and Gamma variants can infect common laboratory mice, leading to increased lung viral load and moderate lung lesions [37,38]. This viral adaptation is partially attributed to changes in key residues at the receptor binding domain (RBD) of S protein, including N501Y, which is also present in mouse-adapted SARS-CoV-2 [39]. 

One of the concerns with the emergence of more infectious variants is the effectiveness of currently used COVID-19 vaccines against the new variants, which may evade vaccine-induced immunity. The cross-protection conferred by vaccination with inactivated WT SARS-CoV-2 against Gamma or Zeta variants using the murine model has not been evaluated. Therefore, we investigated anti-SARS-CoV-2 antibodies titers in individuals vaccinated with the CoronaVac vaccine, an inactivated viral vaccine at 1, 3, and 6 months after the second dose. In addition, we established an animal model to evaluate protective immune response induced by immunization with inactivated WT SARS-CoV-2 against the Gamma and Zeta variants, evaluating for antibody production, tissue viral load, inflammatory cytokine expression, lung histopathological analysis, and serum neutralizing activities. 

## 2. Materials and Methods

### 2.1. Study Participants

**Figure 1:**
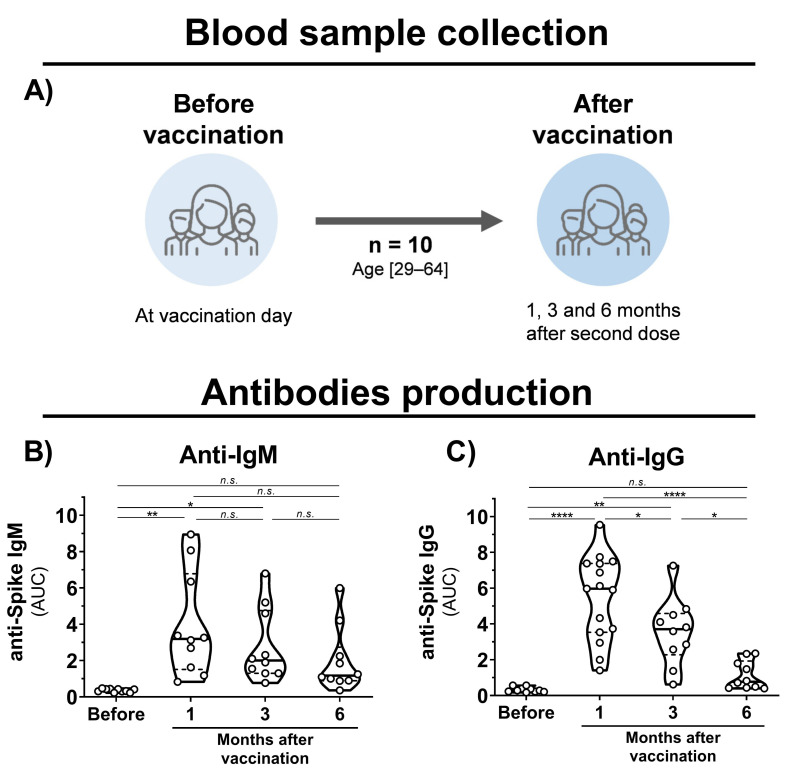
Blood sample collection time points and antibody production in CoronaVac-vaccinated individuals. (**A**) Schematic representation of established cohort and sample collection. (**B**,**C**) Violin plots showing anti-S IgM and IgG levels, respectively, of vaccinated individuals before vaccination and after 1, 3, and 6 months of the second dose. Multiple comparisons were performed by one-way ANOVA with post hoc Tukey’s test. * *p* < 0.05; ** *p* < 0.001; **** *p* < 0.0001. AUC = area under curve; n.s. = not statistically significant; IgG = immunoglobulin G.

Blood samples were collected from healthy volunteers that had been vaccinated with the CoronaVac vaccine (Sinovac Life Sciences, Beijing, China). Eligibility criteria were a minimal age of 18 years old, no history of COVID-19 disease, and CoronaVac vaccination. Volunteers were recruited at Virology Research Center and Clinics Hospital, both from Ribeirão Preto Medical School. The cohort (n = 10) was composed of researchers and medical workers aged 29–64 (average of 44) consisting of 6 males and 4 females (Figure 1A). Samples were collected before the first dose and 1, 3, and 6 months after the second dose.

### 2.2. Viruses and Cells

Three different SARS-CoV-2 lineages were used in this study, the WT (Genbank access MT126808.1), Gamma (P.1) (GISAID: EPI_ISL_2499748), and Zeta (P.2) (GISAID: EPI_ISL_770561), which were isolated from clinical samples in Brazil [23,40]. Viral stocks were propagated in Vero E6 cells (ATCC CRL-1586) cultured with Dulbecco’s Modified Eagle’s Medium (DMEM) (Vitrocell, Campinas, São Paulo, Brazil) containing 1% penicillin/streptomycin and 2% fetal bovine serum (FBS), and maintained at 37 °C and 5% CO_2_. After 3 days postinfection, the cell culture supernatant was collected, clarified by low-speed centrifugation (300× *g* for 10 min), and stored at −80 °C. Viral titration was performed by plaque-forming unit (PFU) assay as previously described [40]. 

### 2.3. Preparation of Inactivated SARS-CoV-2

For immunization, inactivated WT SARS-CoV-2 was prepared on the basis of previously described studies [41]. Briefly, Vero E6 cells were propagated in a T-150 flask, the cell culture supernatant was removed; then, cells were washed twice with phosphate-buffered saline (PBS), inoculated with WT SARS-CoV-2, and incubated for 1 h under gently rocking for viral adsorption. As a negative control, only culture media were used (Mock). After incubation, DMEM with 2% FBS was added, and cells were incubated at 37 °C and 5% CO_2_ until observation of cytopathic effects (>50%). The cell culture supernatant was collected, clarified by low-speed centrifugation, and incubated with β-propiolactone (Sigma-Aldrich, St. Louis, MO, USA) at 0.05% (*v*/*v*) for 20 h at 4 °C under gentle rocking for viral inactivation. The solution was ultracentrifuged at 170,000× *g* for 1 h, and the viral pellet was resuspended in 2 mL of PBS. To hydrolyze residual β-propiolactone, the inactivated virus was incubated for 2 h at 37 °C on a warm bath. For in vitro cell stimulation, viral particles from the cell culture supernatant were heat-inactivated by incubating at 60 °C for 2 h on a warm bath. Total protein concentration was determined by Bradford assay using Quick Start Bradford Dye Reagent (Biorad, Hercules, CA, USA), and samples were kept at −80 °C until usage. To evaluate viral inactivation, samples were inoculated on Vero E6 cells, incubated at 37 °C and 5% CO_2,_ and monitored daily for cytopathic effects. 

### 2.4. Mouse Immunization, Infection, and Sample Collection

Six-week-old female C57BL/6 mice were used for immunization twice, at 0 and 14 days with 10^5^ PFUs of inactivated WT SARS-CoV-2 or Mock with 30% (*v*/*v*) of hydroxide aluminum adjuvant by intramuscular route. Blood samples were collected at 14 and 28 days after initial immunization by facial bleeding. The blood was centrifuged, and the sera were collected for humoral immune response evaluation. For cellular immune response analysis, splenocytes were collected after 28 days of vaccination and stimulated in vitro with SARS-CoV-2 antigens. Some immunized mice were infected with 7 × 10^4^ PFUs of WT SARS-CoV-2, and Gamma or Zeta variants by intranasal route at 28 days after the initial immunization. Three days after the viral infection, blood samples were collected for antibody measurement and neutralizing assays. Mice were euthanized, and the lungs were collected for analysis. The superior right lobe was gently perfused with a 10% formaldehyde solution, kept in the same solution for 7 days, processed using the automated PT05 TS processor (Lupetec, São Carlos, São Paulo, Brazil), embedded in paraffin, sectioned at 4 µm thickness, and stained with Harris’s hematoxylin and eosin (H&E) for histopathological analysis. The left lung was collected in Roswell Park Memorial Institute (RPMI) 1640 Medium (Sigma-Aldrich, St. Louis, Missouri USA) containing 1% penicillin/streptomycin and 5% FBS, and processed for flow cytometry analysis. The middle and inferior right lobes were weighed and homogenized with PBS (1:5 *w*/*v*) using a 5 mm stainless steel bead (Qiagen, Germantown, Maryland, USA) and a TissueLyser LT (Qiagen, Germantown, MD, USA), (50 Hz for 5 min), centrifuged at 10,000× *g* for 10 min, and the supernatant was collected and used for viral load measurement by plaque assay, qRT-PCR, and cytokine quantification. 

### 2.5. Viral Quantification by Plaque Assay 

For plaque assay, one day before infection, 5 × 10^4^ Vero E6 cells were plated in a 48-well plate and cultivated overnight in DMEM containing 10% FBS. On the next day, samples were serially diluted in DMEM from 10^−1^ to 10^−6^, cell culture supernatant was removed, and viral dilutions were inoculated in duplicate. Plates were incubated for 1 h at room temperature under gentle rocking to allow for viral adsorption. Next, 500 µL of prewarmed overlay media (DMEM containing 2% (*w*/*v*) carboxymethylcellulose and 2% FBS) was added to each well, and plates were incubated for 5 days at 37 °C and 5% CO_2_ to allow for plaque formation. Then, cells were fixed for 2 h with 4% formaldehyde solution and stained with 1% naphthol blue-black (Sigma-Aldrich, St. Louis, MO, USA) solution for 1 h for plaque visualization. 

### 2.6. Viral Quantification by RT-PCR

RNA was extracted with TRIzol (Thermo Fisher Scientific, Waltham, Massachusetts, USA) reagent with 20 mg of lung homogenate, according to the manufacturer’s recommendation. For viral RNA quantification, qRT-PCR was performed using TaqMan Fast Virus 1-Step Mix (Applied Biosystems, Waltham, MA, USA) according to the manufacturer’s recommendations. SARS-CoV-2 primers and probe were designed according to a previously reported protocol (REF). They target a 100 bp region from RNA-dependent RNA polymerase gene (*RdRp*) of all three SARS-CoV-2 variants (forward: 5′-GTGAAATGGTCATGTGTGGCGG-3′; reverse: 5′-CAAATGTTAAAAACACTATTAGCATA-3′; and probe: ‘5-FAM-CAGGTGGAACCTCATCAGGAGATGC-BHQ1-3′). Reactions were performed using a StepOnePlus Real-Time PCR system (Applied Biosystems, Waltham, Massachusetts, USA). Each sample was analyzed in duplicate, and results were compared to a standard relative curve using viral stocks from cell culture with a known titer in PFU/mL.

### 2.7. Plaque Reduction Neutralization Assay

To determine the neutralizing activity of mouse and human sera, plaque reduction neutralization assays with a 50% cutoff (PRNT_50_) were performed. Briefly, 5 × 10^4^ Vero E6 cells were plated in a 48-well plate in DMEM containing 10% FBS, and cultivated overnight at 37 °C and 5% CO_2_. Then, serum samples were heat-inactivated at 56 °C for 1 h, serially diluted from 1:5 to 1:160 (human samples) or 1:10 to 1:320 (mice samples) in DMEM, and mixed with 10^2^ PFU of different SARS-CoV-2 variants. As a positive control, media only were used instead of the sample. The virus–serum mixture was incubated for 1 h at 37 °C to allow for antibody–virus-binding complex formation. Subsequently, the cell culture supernatant was removed, and the antibody–virus mixtures were inoculated to each well in duplicate. For viral adsorption, the plates were incubated for 1 h under gentle rocking at room temperature. The residual viral inoculum was removed, and a prewarmed overlay medium was added to each well. To allow for plaque formation, plates were incubated for 4 days (WT) or 5 days (Gamma and Zeta variants) at 37 °C and 5% CO_2_. For plaque counting, cells were fixed for 2 h with a 4% formaldehyde solution and stained with 1% Naphthol blue-black (Sigma-Aldrich, St. Louis, Missouri, USA) solution for 1 h. Neutralization activity was determined by reduction in plaque formation from the sample–virus mixture when compared to the positive control. The area under the curve (AUC) was calculated using Prism v.8.0.2 (GraphPad, San Diego, CA, USA).

### 2.8. Antibody Quantification by ELISA 

Antibody production against SARS-CoV-2 were determined using recombinant spike (S) and nucleocapsid (N) proteins as capture antigens in the ELISA format. ELISA plates were coated overnight with 50 μL of S or N protein diluted at 4 μg/mL in carbonate–bicarbonate pH 9.6 buffer (Sigma-Aldrich, St. Louis, MO, USA) at 4 °C. On the next day, plates were blocked with blocking buffer (PBS-T with 2% [*v*/*v*] bovine serum albumin) for 2 h at 37 °C. Next, serum sample dilutions (twofold diluted from 1:50 to 1:400) were added in duplicate and incubated for 1 h at 37 °C. Then, plates were incubated with antihuman IgG or IgM (Sigma-Aldrich, St. Louis, MO, USA) or antimouse IgG or IgM (Sigma-Aldrich, St. Louis, MO, USA) conjugated to horseradish peroxidase (HRP) for 1 h at 37 °C and revealed with TMB solution (KPL, Boca Raton, FL, USA). The reaction was stopped by adding 1M H_2_SO_4_, and absorbance was measured at 450/620 ɳm with a Multiscan MMC/340 microplate reader (Titertek, San Bruno, CA, USA). The AUC was calculated using Prism v.8.0.2 (GraphPad, San Diego, CA, USA).

### 2.9. Lymphoproliferation Assay

Spleens were collected from C57BL/6 mice 28 days after immunization and processed for lymphoproliferation assay. Briefly, 2 × 10^5^ splenocytes were stimulated with 10 µg of heat-inactivated viral antigens for 5 days at 37 °C and 5% CO_2_. Phytohemagglutinin (Vitrocell, Campinas, São Paulo, Brazil) was used as positive control, and lymphoproliferation was determined using CellTiter-Glo (Promega, Madison, WI, USA) according to the manufacturer’s recommendations. The luminescence signal was normalized with mock stimuli, and the immunized group signal was normalized with the mock immunized group.

### 2.10. Cytokine Quantification from Mouse Lungs

Cytokine profile was determined on the third day after viral infection with the three viral lineages in mouse lungs using the Cytometric Bead Array (CBA) Mouse Th1/Th2/Th17 Cytokine kit (BD Biosciences, Franklin Lakes, NJ, USA). Procedures were performed according to manufacturer instructions, and samples were acquired on BD FACSCanto (BD Biosciences, Franklin Lakes, NJ, USA) and analyzed using FCAP Array v3.0 (BD Biosciences, Franklin Lakes, NJ, USA).

### 2.11. Flow Cytometry

The middle and inferior lobes were collected and digested with collagenase (2.2 mg/mL) and DNAse (0.055 mg/mL) (Roche, Basel, Switzerland). Samples were stained for viability with FVS780 and cell subpopulations with CD45-PE-Cy7 (30-F11), CD3-FITC (145-2C11), CD4-BB700 (RM4-5), CD8-PE (53–6.7), and CD25-APC (PC61). Samples were acquired in FACS Melody (BD Biosciences, Franklin Lakes, New Jersey, USA), and analyses were performed in FlowJo software (BD Biosciences, Franklin Lakes, NJ, USA).

### 2.12. Statistical Analysis

Statistical analysis was conducted using GraphPad Prism 8.0.2 software (GraphPad, San Diego, CA, USA). Grubb’s test was applied to detect and remove possible outliers, and the Kolmogorov–Smirnoff test was used to verify data distribution. Comparison between two distinct groups was determined using an unpaired, two-tailed Student’s *t*-test with a 95% confidence interval. For multiple-group comparisons, one- or two-way analysis of variance (ANOVA) was used, followed by the post hoc test specified in each figure legend. Statistical differences were considered significant when *p* value < 0.05.

## 3. Results

### 3.1. Individuals Vaccinated with CoronaVac Produce Reactive Antibodies against Gamma and Zeta Variants 

**Figure 2:**
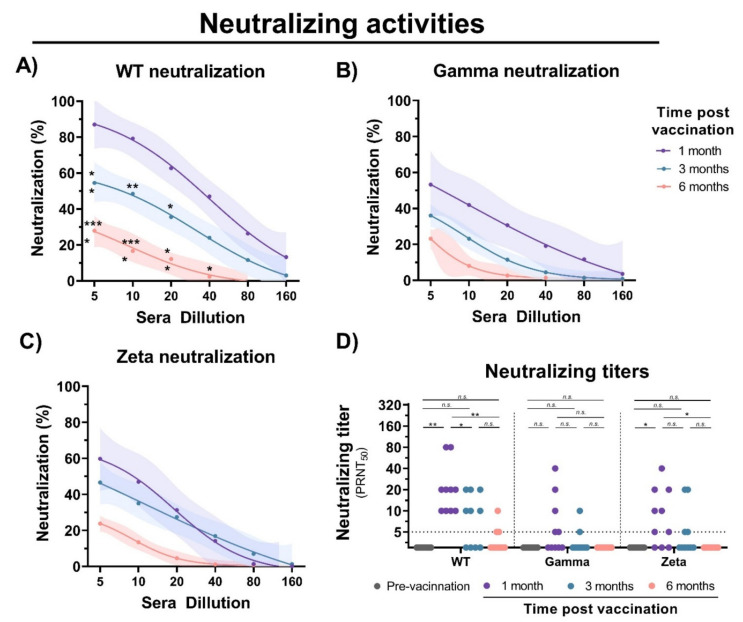
Neutralizing activity of sera from CoronaVac-vaccinated individuals. Serial dilutions of serum samples were prepared, and neutralization activity was evaluated against (**A**) WT SARS-CoV-2, and (**B**) Gamma and (**C**) Zeta variants at 1, 3, and 6 months after second dose. Nonlinear regression curves generated with a maximum of 1000 interactions and 95% confidence interval. Error bars delimited by colors. Multiple comparisons were performed by two-way ANOVA followed by Sidak’s post hoc test. Asterisks, significance difference compared to 1-month group. * *p* < 0.05; ** *p* < 0.001; *** *p* < 0.0001. (**D**) PRNT50 neutralizing titers of WT SARS-CoV-2, and Gamma and Zeta variants using sera from vaccinated individuals collected at different time points, indicated by black (prevaccination), purple (1 month), turquoise (3 months), and red (6 months). Multiple comparisons were performed by one-way ANOVA followed by Tukey’s post hoc test. * *p* < 0.05; ** *p* < 0.001. PRNT50 = 50% plaque reduction neutralization test; n.s.= not statistically significantly; LOD = limit of detection.

To evaluate the immunogenicity of immunization with inactivated SARS-CoV-2, blood samples were collected from individuals before vaccination, and 1, 3, and 6 months after the second vaccine dose with CoronaVac (Figure 1A). Detection of IgM antibodies increased after initial vaccination, but nonsignificant changes were observed after 6 months (Figure 1B). Production of anti-S IgG was observed after 1 month of vaccination; however, a 1.5- and 5-fold decrease in IgG levels was observed after 3 and 6 months, respectively (Figure 1C). Unchanged levels of N-specific IgG and IgM antibodies were observed before and after vaccination. A decrease in neutralizing activities was observed at 1, 3, and 6 months of vaccination with a significant reduction in cross-neutralizing titers for the Gamma and Zeta variants (Figure 2A–D). Collectively, these results demonstrate that vaccination with inactivated SARS-CoV-2 is able to induce S-specific IgG and IgM antibodies, but with a reduction over 6 months, especially in cross-neutralizing titers against the Gamma and Zeta variants.

### 3.2. Mouse Immunization with Inactivated WT SARS-CoV-2 Induces Cross-Reactive Immune Response to Gamma and Zeta Variants 

**Figure 3:**
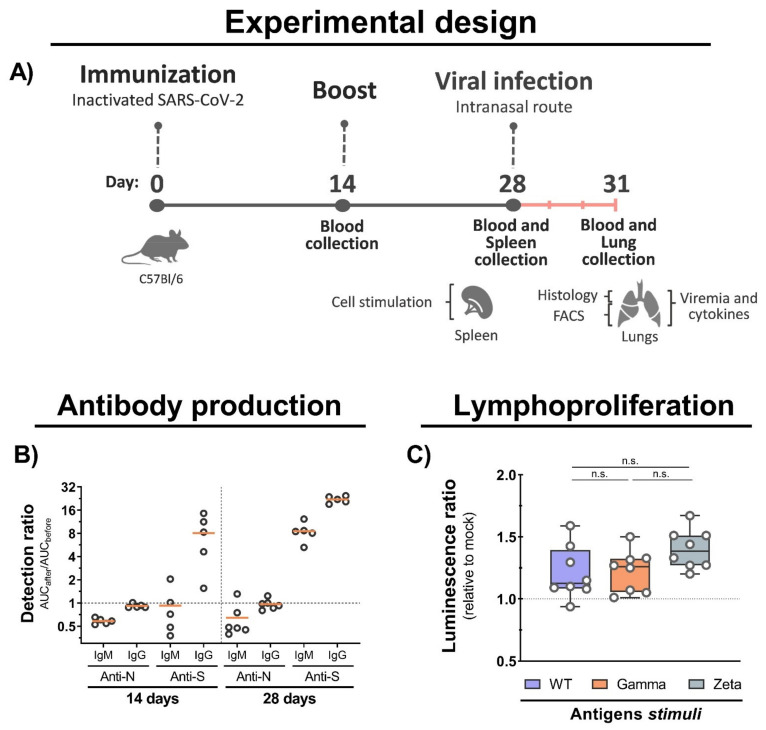
Mouse immunization, antibody production, and cellular immune response. (**A**) C57BL/6 mice were intramuscular immunized with inactivated WT SARS-CoV-2 at days 0 and 14, and intranasally infected with 7 × 10^4^ PFU (WT/Gamma/Zeta) at day 28. Blood serum samples collected at 14 and 28 days after immunization, and 3 days after infection, and used for antibody analysis. Spleen organs collected on day 28 and used for lymphoproliferation assay. Three days after infection, mice were euthanized and the lungs collected for analysis. (**B**) IgM and IgG antibodies production anti-N and anti-S after 14, and 28 days of initial immunization were detected by indirect ELISA. AUC from immunized mice was normalized with AUC from the same mice before infection. (**C**) Lymphoproliferation assay was performed with splenocytes from immunized and nonimmunized mice stimulated for 5 days with 10 μg of inactivated viral antigens of WT SARS-CoV-2, Gamma or Zeta viral lineage. Statistical analysis was conducted by one-way ANOVA followed by Tukey’s post hoc test. n.s. = not statistically significant; AUC = area under curve; IgM = immunoglobulin M; IgG = immunoglobulin G.

To assess the immunogenicity of inactivated WT SARS-CoV-2, we set up a two-dose immunization protocol using C57BL/6 mice. Blood, spleen, and lung samples were collected before and after viral infection (Figure 3A). We observed an increased production of IgG antibodies against the S protein at 14 and 28 days after immunization with an increased ratio of approximately 10- and 30-fold, respectively, and an undetectable production of antibodies to the N protein (Figure 3B). Furthermore, we observed an increased lymphoproliferation of spleen cells from immunized mice after 5 days of stimulation with total viral antigens of the WT, Gamma, and Zeta lineages, but no significant proliferation differences were observed among them (Figure 3C). These results show that immunization with inactivated WT SARS-CoV-2 induces the production of anti-S antibodies, and cross-reactive cellular response to Gamma and Zeta variants.

### 3.3. Immunization with Inactivated WT SARS-CoV-2 Reduces Viral Load and Proinflammatory Cytokine Production in Mouse Lungs after Gamma and Zeta Variant Infection

**Figure 4:**
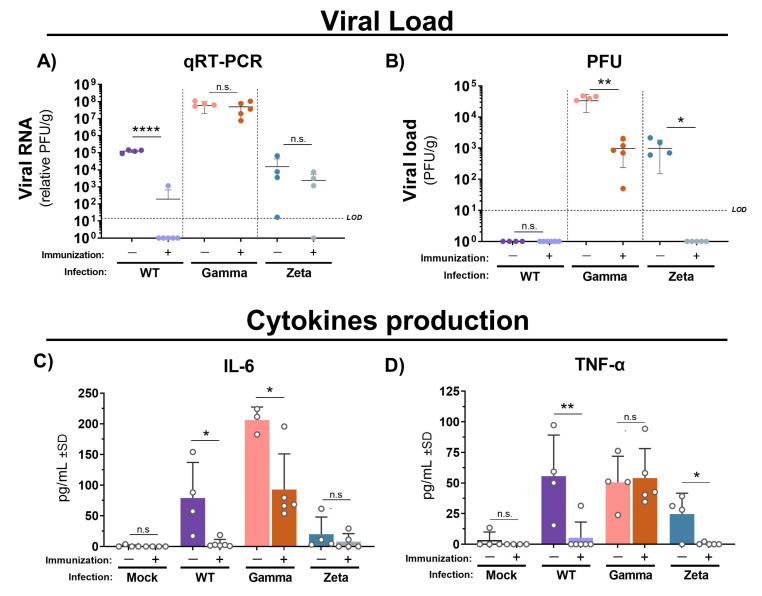
Viral loads and cytokine production in lungs of immunized and nonimmunized mice after infection with WT SARS-CoV-2, Gamma, or Zeta viral lineage. Three days after viral infection, the left lung was weighted and homogenized, and the supernatant was used for viral load determination by (**A**) qRT-PCR and (**B**) PFU. Cytokine production of (**C**) IL-6 and (**D**) TNF-α. Number of mice per group indicated by circles. Unpaired *t*-test performed between groups infected with same viral lineage. * *p* < 0.05; ** *p* < 0.01; **** *p* < 0.0001). LOD = limit of detection; qRT-PCR = quantitative real-time polymerase chain reaction; PFU = plaque forming unit; g = gram of tissue; n.s. = not statistically significant.

Next, we assessed the cytokine profile and protective efficacy of immunization with inactivated WT SARS-CoV-2 in C57BL/6 mice against Gamma and Zeta variant infection. Viral RNA levels quantified by qRT-PCR were practically undetectable in lungs from mice immunized and infected with WT SARS-CoV-2, but no significant changes were observed in either immunized or nonimmunized groups after infection with the Gamma or Zeta variant (Figure 4A). However, viral load in mouse lungs measured by plaque assay was significantly reduced in immunized mice after infection with Gamma or Zeta variants (Figure 4B). IL-6 production in immunized mice was reduced to almost undetectable levels after the infection with the WT lineage, and reduced twofold after infection with the Gamma variant, while the Zeta variant induced less production of IL-6 and nonsignificant differences between immunized and nonimmunized mice (Figure 4C). TNF production in immunized mice was reduced 10-fold after infection with the WT or Zeta variant; no significant changes were observed in immunized or nonimmunized mice after infection with the Gamma variant (Figure 4D). Levels of IL-10, IL-4, IL-2, IL-17, and IFN-γ were under the detection limit. 

### 3.4. Immunization Reduces Lung Injury in Mice after Infection with Gamma and Zeta Variants

**Figure 5:**
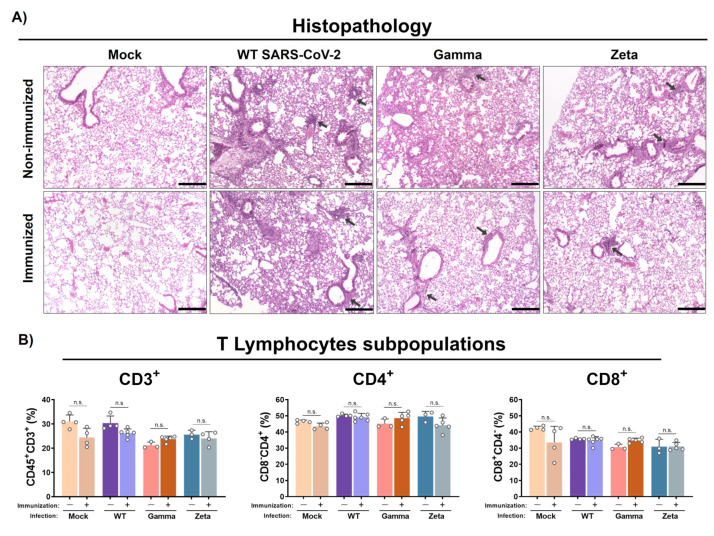
Histopathology and T lymphocyte subpopulation analysis from mouse lungs. Lungs from immunized and nonimmunized mice were collected after 3 days of infection. (**A**) Superior right lobe processed and stained with H&E. (**B**) Middle and inferior lobes collected and evaluated for recruitment of T lymphocyte subpopulations. n = 3 for nonimmunized groups and n = 5 for immunized groups. Black arrows, mixed inflammatory infiltrates. Scale bar = 200 µm. Statistical analysis conducted by one-way ANOVA followed by Tukey’s post hoc test. n.s. = not statistically significant.

Histological analysis showed that WT SARS-CoV-2 infection induced mild congestion and a moderate mixed inflammatory infiltrate (mononuclear cells and scarce neutrophils) in the lungs of immunized or nonimmunized mice (Figure 5A). However, nonimmunized mice infected with the Gamma variant showed more representative lesions, characterized by a diffuse alveolar thickening due to a moderate mixed inflammatory infiltrate when compared to the immunized group, which showed just a mild peribronchovascular inflammatory infiltrate. On the other hand, the nonimmunized group infected with the Zeta variant exhibited a reduced inflammatory infiltrate in the alveolar septum, more evident only in the peribronchovascular area, while the immunized group presented a clear reduction with mild inflammatory focus. Control groups showed preserved lung architecture. No significant changes were observed for CD3^+^, CD4^+^, and CD8^+^ T lymphocyte subpopulations percentages in immunized or nonimmunized mouse lungs after 3 days of infection (Figure 5B).

### 3.5. Antibody Production and Neutralization Activities after Viral Infection

**Figure 6:**
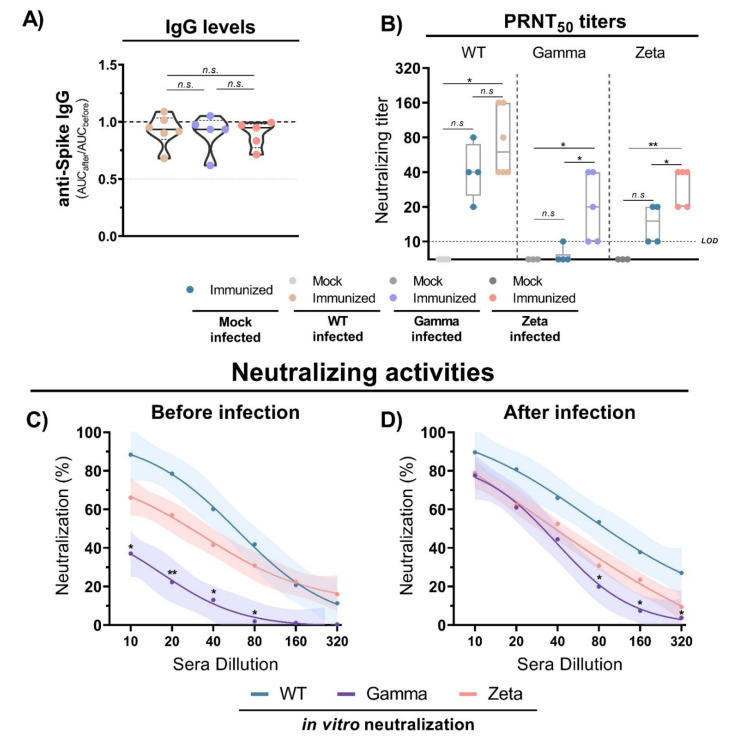
Antibody titers and neutralization activity in mice immunized with WT SARS-CoV-2 and infection with different viral lineages. (**A**) Anti-S IgG level ratio from immunized mice sera before and after viral infection with WT SARS-CoV-2, Gamma or Zeta lineages, normalized. N= 5–6 animals/group (indicated on figure). Multiple comparisons performed by ordinary one-way ANOVA followed by Tukey’s post hoc test. (**B**) Neutralizing titers in PRNT50 of immunized (n = 4) and nonimmunized (n = 3) mouse sera before and after viral infection with WT SARS-CoV-2 (n = 6), Gamma (n = 5), or Zeta (n = 5) lineages. (**C**,**D**) Neutralizing profile against WT SARS-CoV-2, Gamma, or Zeta lineages from immunized mouse sera before (n = 4) and 3 days after (n = 6, for each group) viral infection. Nonlinear regression curves generated with a maximum of 1000 interactions and 95% confidence interval. Error bars delimited by colors. Multiple comparisons performed by two-way ANOVA followed by Tukey’s post hoc test. * *p* < 0.05; ** *p* < 0.001. AUC = area under curve; PRNT50 = 50% plaque reduction neutralization test; LOD = limit of detection; n.s. = not statistically significant.

The evaluation of anti-S IgG antibody production 3 days after infection with WT SARS-CoV-2, and Gamma and Zeta variants in immunized mice showed no significant differences among the groups (Figure 6A). However, the viral infection induced an increase in neutralizing titers for the three mouse groups (Figure 6B). Sera from immunized mice before the infection showed reduced neutralizing capacity against the Gamma variant compared to WT lineage (Figure 6C). However, 3 days after viral infection, the neutralizing profile rapidly shifted, and the three mouse groups showed a similar profile of neutralizing activity (Figure 6D). These results indicate that anti-S IgG antibody titers do not significantly change after viral infection, but neutralizing activities rapidly increase, demonstrating a fast cross-reactive immune response against the Gamma and Zeta variants.

## 4. Discussion

In this study, we evaluated immune response after immunization with CoronaVac, an inactivated SARS-CoV-2-based vaccine. In agreement with a clinical trial study of CoronaVac, we observed an induction of similar levels of neutralizing activity with the production of anti-S IgG antibodies after 1 month of vaccination. However, when compared to other commercially available vaccines, such as BNT162b2 (Pfizer), ChAdOx1 (AstraZeneca), and Ad26.COV2.S (Johnson & Johnson), the neutralizing titers were apparently lower [21,42,43,44]. We observed a significant decrease in IgG levels in the months after vaccination. This result was expected considering that inactivated vaccines induce a weaker immune response due to antigenicity loss during inactivation. A recent preprint study suggested that a third dose of CoronaVac would be recommended to efficiently recall specific immune responses against SARS-CoV-2 [45]. In agreement, we showed that neutralizing activity against WT SARS-CoV-2 is considerably reduced over the course of 6 months in recipients of CoronaVac. A similar pattern of neutralization decrease was observed against the Gamma and Zeta variants, but with a significant reduction from the first month after vaccination, probably due to immune evasion of the variants [28,30].

Previous studies described that immunization with inactivated SARS-CoV-2 induces a robust production of antibodies and cellular immune response [21,46,47]. Likewise, we showed that mice immunized with inactivated WT SARS-CoV-2 produced anti-S IgG antibodies. Furthermore, mouse spleen cell stimulation with inactivated viral antigens induced lymphoproliferation with no significant differences among different viral antigens, suggesting the existence of cellular immune response to conserved epitopes. These results agree with previous reports showing the production of anti-S IgG antibodies in immunized mice within 1 to 6 weeks, and undetectable differences in lymphocyte activation with viral antigens from different variants [14,31].

High viral load in humans was correlated with increased disease severity and a higher risk of death by COVID-19 [48,49]. In our study, we detected viral RNA in the lung tissue from mice infected by any of the three SARS-CoV-2 lineages. However, only the WT-infected group showed a significant reduction in SARS-CoV-2 RNA levels after immunization. On the other hand, viral load measured by plaque assay was detected only in mice infected with the Gamma or Zeta variant, indicating different susceptibility degrees of C57BL/6 mice to infection. The discrepancy between the detection of viral RNA and viable virus indicates the existence of conservative factors, such as phagocytic cells, which can conserve the viral genetic material in lung tissue for longer periods after infection, even in the absence of viable virus. The viral load from immunized mice showed nearly 2-log reduction and undetectable levels upon infection with the Gamma and Zeta variants, respectively, indicating the induction of protective immunity.

A key characteristic of severe COVID-19 is the dysregulation of inflammatory pathways with the increased production of cytokines such as interleukine-6 (IL-6) and tumor necrosis factor-α (TNF-α) [50,51]. In our study, infection with WT SARS-CoV-2, and Gamma and Zeta variants induced IL-6 and TNF production in nonimmunized mice and variable expression levels according to lineage, reinforcing the different susceptibility of mice to viral infection. Increased levels of IL-6 and TNF were significantly associated with the severity of lung injury in COVID-19 pneumonia. In addition, circulating IL-6 was the independent predictor of lung-injury severity [52]. Consistent with more intense inflammation observed in nonimmunized mice infected with the Gamma variant, we found higher levels of IL-6 in this mice group. Undetectable levels of IL-6 and TNF-α, consistent with a reduction in the pulmonary inflammatory infiltrate, showed that immunization was effective in mice infected with WT SARS-CoV-2 or the Zeta variant. Although less effective for the Gamma variant, IL-6 levels showed a significant reduction after immunization. Collectively, these data suggest that immunization with inactivated WT SARS-CoV-2 virus is able to induce a cross-protective immune response against Gamma and Zeta variant infection. 

Previous studies demonstrated that the Gamma variant can escape the serum neutralizing antibodies of convalescent patients or individuals vaccinated with Oxford-AstraZeneca, Pfizer/BioNTech, or CoronaVac vaccines [28,30]. Here, we showed that, although neutralizing antibody titers were lower for the Gamma and Zeta variants before infection, there was a significant increase as early as 3 days after viral infection, reaching comparable levels to those observed for WT SARS-CoV-2. This induction of neutralizing antibody production against SARS-CoV-2 variants suggests the existence of cross-reactive immunological memory, which is triggered to rapidly respond against the new viral infection.

In agreement, previous reports described that fully vaccinated individuals with inactivation-based vaccines that were subsequently infected by different SARS-CoV-2 variants (vaccine breakthrough) produce high levels of humoral response and serum neutralizing activities, indicating a probable protective immune recall induced by the infection [53,54,55]. In this context and considering our data in the murine model, fully vaccinated individuals with inactivated WT SARS-CoV-2 who had been infected with the Gamma or Zeta variant may rapidly cross-respond to viral infection and produce efficient neutralizing antibodies, which may be critical for protection against COVID-19. This idea is further supported by a recent study that reported two cases of SARS-CoV-2 infection by the Gamma variant in elderly individuals who received CoronaVac but developed only mild symptoms [56].

Taken together, vaccination with inactivated WT SARS-CoV-2 virus is able to induce immunological cross-protection against the Gamma and Zeta variants. Individuals vaccinated with CoronaVac produce elevated levels of anti-S antibodies with significant decay over time. Therefore, it is possible that booster vaccine doses may be necessary to ensure adequate population protection and pandemic control, and further efforts to improve vaccine immunogenicity would be greatly valuable. Immunized mice that had been infected with the variants showed reduced pathological outcomes with a rapid increase in serum neutralizing activity, suggesting the existence of cross-reactive immunological memory to conserved epitopes. Thus, a continuous elucidation of the immunological mechanisms and clinical outcomes of convalescent and vaccinated individuals against new variants is extremely important as a countermeasure against COVID-19.
